# 

**Published:** 1878-12

**Authors:** 


### LISTS OF SOCIETIES.

In the January number of the REGISTER, and in subsequent numbers, their will only be given a list of the state dental societies, and the American Dental Association, and such others as may have sufficient interest to promptly furnish the matter for a correct statement.

It is desirable to have the names of officers, the time and place of meeting, also the number of active members.

The state societies are of more importance, in some respects at least, than those of a more local character.

In order that this list be as complete as possible, it will be necessary for the secretaries of the different societies to send to the Editor of the REGISTER, at the earliest possible moment, the data indicated in this notice.

### CORRECTION.

The article given in the November number of the Register on, "*Nævous, or the Effect of Dental Operations upon the Female During the Period of Utero-Gestation,*" beginning on page four hundred and fifty-six, was through an oversight attributed to Dr. E. S. Chisholm, of Tuscaloosa, Ala., as the author, instead of Dr. S. M. Prothro, of Chattanooga, Tenn. With whom the responsibility rests for this, I can not now decide, the copy not being at hand. The inadvertence is a matter of regret, but it is to be hoped that this correction will meet the eye of every one who reads the article; which is a most excellent one, and I trust the Register will have more from the same pen, and there shall be no mistake in credit next time.

It is not amiss to suggest here that the writer of any article for publication, except it be a mere matter of correspondence, should place his name at the head of the article; that would obviate such a mistake as that here referred to.

### VIRGINIA STATE DENTAL SOCIETY.

The annual meeting of this society will be held in Richmond, Va., Tuesday, December 10th.

CINCINNATI, O., July 13, 1878.

SPENCER & CROCKER,

Ohio Dental Depot.

DEAR SIRS:—Two months or more ago, Dr. W. S. How attached to my S. S. White engine the Engine Elbow invented by him. From the first I regarded it as an improvement. Since replacing the original with a more perfectly made elbow of your manufacture, I am more and more convinced that it is of decided advantage in the working of the engine. It seems to bring the head of the standard towards the front of the patient, and thus secures the greatest freedom of movement in the hand piece without having to flex the cable to the degree required heretofore. There is consequently less friction of the cable against the sheath, and greater steadiness in the running of the burs and disks. I have also found that with its use, the liability of the cable to break is greatly lesssened. Yours truly,

H. A. SMITH.

### VULCANITE *vs.* METAL AS COVERING FOR ROOF OF MOUTH IN UPPER PLATES.

BY W. C. DUNCAN, D. D. S., CINCINNATI.

All dentists who have made rubber plates to any considerable extent, can readily call to mind cases by no means rare, of patients making serious complaints of a heated condition of the mouth, particularly of the mucous membrane in some cases of such an aggravated character as to cause them to abandon the plates. It can hardly admit of a doubt that the non-conductive nature of rubber is the primary cause of the feverishness. With those who are troubled in this way the feeling may vary in degree from a slightly tantalizing one to a severity so great as to debar the wearing of the plate. Two cases I will relate.

Sister ——, in one of the religious orders of this city, a lady of superior attainments in general physics, for four years had been wearing an upper vulcanite plate with satisfaction, at the end of that time a nameless unpleasant feeling developed itself in the roof of the mouth. There was nothing in the appearance to indicate the cause of the sensation; after enduring the annoyance for some time, and resorting to various expedients without benefit, the thought occurred to me that the substitution of metal as a base, (conduction of heat and electricity is one of the characteristics of the metals) might overcome the trouble. I named it to her. She responded at once, informing me that she had laterally observed an excess of electricity in her system. The substitution of a gold plate met all the indications.

Mrs. S——, was the most notable case in my experience. She obtained an upper plate of vulcanite, having two sisters much pleased with plates of that kind. She returned the day after the teeth were inserted, stating, the only way she was enabled to keep the plate in her mouth was by constantly filling her mouth with cold water in order to allay the extreme feverishness she experienced. After canvassing the case I became satisfied her case required metal. I swaged a silver plate which I gave her to wear as a test. The reaction was complete, thus proving that the absence of conductivity in the rubber was the cause of the heated condition of the mouth. Teeth were afterwards added to the plate.

My attention has recently been called to an ingenous device called a *Palate Cooler*, which can be readily inserted in rubber or celluloid plates, and which it seems to me will effectually prevent the heated condition of the mouth above mentioned, and at the same time preserve the perfect fit obtainable with plates made of vegetable material.

### NEW CHAIRS.

We are receiving orders for the Wilkerson Chair, also the S. S. White Pedal-Lever Chair. See illustration in this No. Register.

### THE DENTAL COLLEGE

OF THE

### UNIVERSITY OF MICHIGAN.

THE FOURTH ANNUAL SESSION of this Institution will commence on the 1st of October and close on the last Wednesday of March, thus making a course of six months. The regular course of instruction commences at once, and will proceed through the term, with the customary holiday vacation.

The students in this department will receive instructions in Anatomy, Physiology, Pathology, Chemistry, Meteria Medica, Therapeutics and Surgery, from the professors of their respective branches in the *Department of Medicine and Surgery* of the University, when lectures commence, and continue the same as with the Dental College.

### FACULTY OF THE DENTAL DEPARTMENT.

J. B. ANGELL, LL. D., President.

J. TAFT, D.D.S., Professor of Principles and Practice of Operative Dentistry.
J. A, WATLING, D.D.S., Professor of Clinical and Mechanical Dentistry.
W. H. DONENEE, Demonstrator of Mechanical Dentistry.

It is desirable that students should be present at the opening of the session, as the regular course of instruction begins at that time. Seats in the lecture-room are assigned by selection to students in the order of registration on the steward's books; and each student is expected to occupy during the session such seat as he may select. Students, on arriving in Ann Arbor, should call at the steward's office.

### CONDITIONS OF GRADUATION.

The candidate must be twenty-one years of age. He must furnish evidence of good moral character.

He must devote three years in the study of his profession, He must attend two full courses of lectures in the Dental College, or one course elsewhere and the last one here, and we recommend that he attend three courses regularly.

He must sustain an examination satisfactory to the Faculty in all the branches taught.

A graduate of the Medical College may enter the senior class, and, if found qualified, may graduate after one year has been devoted tc the study of dentistry.

### FEES AND EXPENCES.

The fees, which must be paid in advance, are as follows:

MATRICULATION FEE.—Residents of Michigan, $10.00; non-residents, $12.00.

ANNUAL DUES·—Residents of Michigan, $20.00; non-residents, $25-00.

GRADUATION FEE.—For all alike, $10.00. The admission fee is paid but once, and entitles the students to the privileges of permanent membership in any department of the University, The annual tax is paid the first year and every year after.

For further particulars, address the Dean of the Dental College, Ann Arbor, Michigan.

Jan-6t, J. TAFT, DEAN.

*American Dental Association*—Meets on the first Tuesday in August, 1878, at Niagara Falls. Pres. F. H. Rehwinkel, Chillicothe, Ohio; Cor. Sec. J. H. McQuillen, Philadelphia; Rec. Sec. M. S. Dean, Chicago.

*American Dental Convention*—Meets at Niagara Falls, first Tuesday in August, 1878. Pres. J. Taft, Cincinnati, Ohio; Rec. Sec. A. Tees, Philadelphia, Pa.

List of Societies Represented in the American Dental Association:

*American Academy of Dental Surgery*—Meets in New York 1878. Pres. Geo. H. Perine; Rec. Sec. N. M. Beckwith.

*American Academy of Dental Science*—Meets at Boston, Mass. Pres. Dr. D. M. Parker; Sec. W. L. Tucker, of Boston Cor. Sec. Geo. T. Moffat.

*Baltimore Dental Society*—Pres. C. T. Brockett; Rec. Sec. F. F. Drew.

*Brooklyn Dental Society*—Meets semi-monthly. Pres. O. E. Hill; Rec. Sec. C. P. Crandell.

*Buffalo Dental Society*—Meets second Monday of each month, at Buffalo. Pres. S. A. Freeman; Rec. Sec. G. C. Daboll.

*Boston Dental Institute*—Meets first Monday of each month. Pres. D. G. Williams, Boston; Cor. Sec. D. S. Dickerman.

*California State Dental Association*—Meets first Tuesday in June, 1878. Pres. Wm. B. Kingsbury; Sec. H. J. Planteaux.

*Chicago Dental Society*—Meets monthy at Chicago. Pres. C. R. E. Koch; Rec. Sec. D. B. Freeman.

*Connecticut Valley Dental Association*—Meets semi-annually, second Tuesday of June and October. Pres. L. C. Laylor, Hartford, Conn.; Rec. Sec. C. T. Stockwell Springfield, Mass. Next Meeting in Springfield, Mass., October 17, 1877.

*Connecticut State Dental Society*—Pres. E. T. Payne; Rec. Sec. Geo. L. Parmelee, Hartford. Semi-annual meeting in October in Hartford.

*Charlestown Dental Association*—Meets second Tuesday of each month. Pres. T. F. Chupein; Sec. and Treas. B. A. Muckenfuss.

*Central Pennsylvania Dental Association*—Meets at Huntington, Jan. 1878. Pres. E. J. Green; Sec. W. B. Miller.

*Cincinnati Dental Society*—Meets first and third Tuesday of each month. Pres. A. Berry; Sec. A. Downing.

*Central Ohio Dental Society*—Meets at Crestline, on the second Monday in April, 1877. Pres. M. DeCamp; Sec. N. J. Cressinger.

*Dental Society of the State of Maryland and District of Columbia*—Meets annually. Next meeting at Deer Park, on the second Tuesday in August, 1877. Pres. R. F. Hunt, of Washington, D. C.; Sec. H. C, Thompson.

*Eastern Indiana Dental Association*—Meets semi-annually, first Tuesday in May, and last Tuesday in October; Pres. J. W. Ellis, New Castle; Sec. M. H. Chappell, Knightstown, Ind. Place of meeting, Cambridge City.

*East Tennesee Dental Association*—Meets annually. Next meeting will be held at Cleveland, Tenn., on the Third Wednesday of Sept , 1877. Pres. James Carson; Sec. J. U. Lee, of Chattanooga.

*Georgia State Dental Society*—July 30th, 1878. Pres. M. H. Thomas, Monroe, Ga. Rec. Sec. J. A. Chopple, Lagrange, Ga. Cor. Sec. Dr. M. S, Jobson, Perry, Ga.

*Harris Dental Association*—Meets quarterly. Next Meeting at Columbia, Pa., first Thursday in August. Pres. J. D. Heiges; Sec. W. N. Amer, Lancaster, Pa.

*Hudson Valley Dental Association*—Meets monthly at Troy, N. Y. Pres. L. C. Wheeler; Rec. Sec. S. G. Andres. Cor. Sec. S. D. French.

*Illinois State Dental Society*—Meets annually. Pres. C. R. E. Koch, Chicago; Sec. E. D. Swain, Chicago. Next place of meeting, Rockford, second Tuesday in May, 1878.

*Indiana State Dental Society*—Meets next year at Indianapolis, on the last Tuesday of June, 1878. Pres. T. S. Hacker, Indianapolis ; Sec. J. A. Turner, Franklin.

*Iowa State Dental Society*—Meets annually. Next meeting at Iowa City on the second Tuesday of May. Pres. G. S. Shattuck, Belle Plane; Rec. Sec. E. E. Hughes of Newton; Cor. Sec. I. P. Wilson, Burlington.

*Kentucky State Dental Assseiation*—Meets annually first Tuesday in June. Next meeting in Covington. Pres. A. H. Smith, of Richmond; Sec. F. Peabody of Louisville.

*Kansas State Dental Association*—Pres. J. D. Patterson; Sec. Wm. H. Shulze, of Atchison. Next meeting will be held at Lawrence, first Tuesday in May, 1878.

*Lake Erie Dental Association*—Meets annually. Pres. C. B. Ansart, Oil City, Penn. Sec. C. D. Elliot, Franklin, Penn. Next meeting first Tuesday of May, 1878.

*Mad River Dental Association*—Meets at Xenia on the first Tuesday in April, and October. Pres. R. Corson, Middletown; Sec. E. Betty, Xenia.

*Maine Dental Society*—Meets in August, at Thomaston. Pres. E. J. Roberts, Augusta. Sec C. E. Hussey, Biddeford.

*Massachusetts Dental Society*—Meets monthly. Pres. John T. Codman, Boston; Rec. Sec. G. F. Grant, Boston; Cor. Sec. T. N. Chandler, Boston.

*Michigan State Dental Association*—Meets annually. Next meeting at Ann Arbor, March 26, 1878. Pres. R. G. Thomas, Detroit; Sec. and Treas. E. S. Holmes, Grand Rapids.

*Mississippi Valley Dental Association*—Meets annually at Cincinnati, on first Wednesday in March, 1878. Pres. A. O. Rawls, Lexington, Ky.; Rec. Sec. Wm. Taft, Cincinnati.

*Mississippi State Dental Association*—Will meet third Wednesday of August. 1878, at Winona. Pres. A. H. Hilzheim, Jackson; Sec. M. C. Marshall.

*Minnesota Dental Association*—Meets annually. Meets May 26. 1878 at Winona. Pres. W. F. Lewis; Sec. C. M. Bailey, Minneapolis.

*Missouri Dental Association*--Meets annually on the first Tuesday of June. Next meeting at Sweet Springs. Pres. G. W. Tindall; Sec. W. H. Eames, of St. Louis.

*Missouri Valley Dental Association*—Meets on the fourth Tuesday in July, 1876, in Omaha, Neb. Pres. J. W. Chadduck; Sec. and Treas. F. M. Shrirer, Tabor, Iowa.

*Merrimac Valley Dental Association*—Meets on the 3d Thursday of May, 1878, in Boston. Pres. C. W. Davis, New Bedford Mass.; Rec. Sec. W. E. Riggs, Lawrence, Mass.; Cor. Sec. D. W. Edgerly, Farmington, Mass.

*Memphis Dental Society*—Meets on the first Tuesday of each month. Pres. W. M. Arrington; Sec. V. F. Elliot.; Treas. C. L. Harris.

*Nashville Dental Association*—Meets on the second Tuesday of each month. Pres. R. R. Freeman; Sec. L. G. Noel, Nashville.

*Nebraska State Dental Society*—Meets annually. Next meeting July 23, 1877, at Fremont. Pres. S. H. King, Lincoln. Sec. W. S. Roseman, Fremont.

*New Jersey State Dental Society*—Meets annually. Next meeting at Long Branch, on the second Tuesday in July, 1877. Pres. T. B. Welch, Newark; Sec. C. A. Meeker, Newark.

*New Hampshire Dental Society*—Pres. D. W. Eagerly, Farmington; Sec. C. W. Clerment, Manchester.

*New Haven Dental Society*— Meets on the first Wednesday of each month at New Haven. Pres. J. T. Metcalf; Rec. and Cor. Sec. C. L. Smith, of New Haven.

*New Orleans Dental Society*—Meets on the first Thursday of each month. Pres. W. S. Chandler; Rec. Sec. F. J. Knapp.

*Northern Ohio Dental Association*—Meets annually. Next meeting at Elyria, on the second Tuesday in May, 1878. Pres. J. W. Lyder, Akron, O. Sec. J. F. Siddall, Oberlin.

*Northern Iowa Dental Association*—Meets annually, on the second Tuesday in June, 1873. Next Meeting at Waterloo. Pres. J. M. Abbot, Lancaster; Rec. Sec. A. V. Eaton, Anamosa; Cor. Sec. A. R. Mason, Waterloo.

*North Carolina Dental Association*--Meets annually. Next meeting at Charlotte, first Tuesday in Sept., 1878. Pres. T. W. Hunter, Salem; Sec. J. H. Crawford, Raleigh.

*North Carolina State Dental Society*—Meets annually. Next meeting at Raleigh, first Wednesday Sept., 1877. Pres. V. E. Turner; Sec. D. A. Robinson.

*Ohio College Dental Association*—Meets annually, February 25, at Cincinnati. Pres. N. G. McKellops, St. Louis, Mo; Rec. Sec. H. A. Smith, of Cincinnati.

*Ohio State Dental Association*—Meets annually at Columbus, first Tuesday of December, 1878. Pres. J. C. Whinnery, Salem; Rec. Sec. A. F. Eminger, Columbus.

*Odontological Society of Pennsylvania*—Meets on the first Wednesday of each month, at 8 o'clock p. m., in the Philadelphia Dental College. Pres. F. M. Dixon; Rec. Sec. E. L. Hewitt.

*Odontological Society of New York City*—Meets the second Tuesday of each month. Pres. A. L. Northrup; Rec. Sec. Wm. Jarvie, Jr.

*Old Colony Dental Association*—Meets quarterly. Pres. E. L. Hathaway; Rec. Sec. L. W. Puffer, North Bridgewater, Mass.

*Ohio Valley Dental Association*—Meets annually, in N. E. Ky., this year in Paris, Ky., Tuesday, September 9th.

*Oregon State Dental Society*—Meets annually. Pres. J. R. Cardwell, Portland; Rec. Sec. G. N. Chance, Portland.

*Pennsylvania State Dental Society*—Meets next in Minnequa Springs, second Tuesday of July, 1877. Pres. J. S. King; Rec. Sec. S. H. Guilford; Cor. Sec. M. H. Webb.

*Pennsylvania Association of Dental Surgeons*—Meets monthly in Philadelphia. Pres Robert Henry; Sec. Jos. Pettit.

*Pittsburg Dental Society*—Meets second Tuesday of each month. Pres. H. W. Arthur, of Alleghany; Sec. E. C. Diehl, Pittsburg.

*San Francisco Dental Association*—Meets monthly. Pres. Alex. Warner. Vice Pres. R. F. Rall; Cor. Sec. S. E. Goe; Rec. Sec. L. L. Dunbar.

*St. Louis Dental Society*—Meets monthly. Pres. A. H. Fuller; Sec. N. C. Stark.

*Susquehanna Dental Association*—Meets annually. Pres. J. N. Johnson; Rec. Sec. E. B. Long. Meets November 14, 1877, at Wilkesbarre.

*Society of Dental Surgeons of the City of New York*—Meets semi-monthly in New York. Pres. B. F. Franklin; Sec. J. S. Latimer; Cor. Sec. E. H. Burras.

*Southern Dental Association*—Meets annually. Next meeting at Deer Park, August 14, 1877. Pres. J. S. Cobb, Nashville, Tenn.; Rec. Sec. E. E. Chisholm, Tuscaloosa, Ala.

*South Carolina State Dental Association.*—Meets annually. Pres. G. F. S. Wright, Columbia; Sec. T. F. Cheupein, Charleston; Cor. Sec. J. S. Thompson, Abbeville. Next meeting at Columbia, the first Tuesday of June, 1877.

*Tennesee Dental Association*—Meets semi-annually. Next meeting at Nashville, fourth Wednesday in June, 1878. Pres. L. G. Noel; Rec. Sec. H. E. Beach; Cor. Sec. J. C. Ross.

*Virginia State Dental Association*—Meets in Richmond, December the 14. Pres. W. W. H. Thackston, Farmville; Sec. G. F. Keesee, Richmond.

*Washington City Dental Society*—Meets monthly. Pres. J. C. Smith; Sec. J. B. TenEyck.

*Wisconson State Dental Association*—Meets annually. Next meeting at Milwaukee, second Tuesday of July, 1877. Pres. R. S. Wells, Sparta; Sec. M. T. Moore, LaCrosse.

### State and District Dental Societies of State of New York.

*New York State Dental Society*—Meets at Albany on the second Wednesday of July next. Pres. W. C. Barrett, Warsaw, N. York.; Cor. Sec. S. B. Palmer; Rec. Sec. S. S. A. Freeman, Buffalo.

*First District*—Meets second and fourth Wednesday evenings of each month. Annual meeting in New York, first Tuesday in June. Pres. J. S. Latimer, New York; Rec. Sec. F. M. Odell, New York.

*Second District*—Annual meeting in Brooklyn, first Monday in April. Pres. C. D. Cook; Rec. Sec. G. L. Mason, Brooklyn; Cor. Sec. W. S. Elliott. The meetings are held quarterly, second Thursday in January, April, July, and October.

*Third District*—Meets semi-annually on the second Tuesday in January and July. Annual meeting at Albany on the second Tuesday in January. Pres. S. P. Welsh.

*Fourth District*—Meets semi-annually. Meets annually at Saratoga on second Tuesday in June. Pres. C. A. Elmore, Ft. Edward; Rec. Sec. E. S. Pearsall, Saratoga.

*Fifth District*—Meets semi-annually. Annual meeting at Syracuse, second Tuesday in June. Pres. F. D. Nellis, Syracuse; Rec. Sec. J. S. Marshall, Syracuse; Cor. Sec. A. B. Cowles, Rome.

*Sixth District*—Meets semi-annually. Annual meeting at Binghampton, on second Tuesday in June. Pres. Frank B. Darby, Oswego; Rec. Sec. F. E. Perry, Norwich; Cor. Sec. A. M. Holmes, Morrisville.

*Seventh District*—Meets semi-annually. Annual meeting at Rochester, first Tuesday in June. Pres. H. S. Miller; Rec. Sec. J. E. Line, Rochester.

*Eighth District*—Meets semi-annually. Annual meeting on second Tuesday in May. Pres. G. C. Daboll; Rec. Sec. S. A. Freeman; Cor. Sec. T. G. Lewis, Buffalo.

*The Seventh and Eighth District Societies*—Hold a union meeting on the last Tuesday of October, alternately at Buffalo and Rochester.

*The Ohio State Board of Dental Examiners* will meet in Columbus on the first Tuesday of December, 1878, at 12 o'clock, M. Pres. J. Taft; Sec. W. P. Horton.

### EXCHANGES.

ST. LOUIS MEDICAL AND SURGICAL JOURNAL.
BOSTON MEDICAL AND SURGICAL JOURNAL.
THE PACIFIC MEDICAL AND SURICAL JOURNAL, SAN FRANCISCO.
PHILADELPHIA MEDICAL AND SURGICAL JOURNAL.
CINCINNATI MEDICAL ADVANCE.
CINCINNATI LANCET AND OBSERVER.
DENTAL COSMOS, PHILADELPHIA.
LADIES' REPOSITORY, CINCINNATI.
CHICAGO MEDICAL EXAMINER.
THE DETROIT REVIEW OF MEDICINE AND PHARMACY.
MEDICAL RECORD, N. Y.
NASHVILLE JOURNAL OF MEDICINE AND SURGERY.
AMERICAN JOURNAL OF DENTAL SCIENCE, BALTIMORE.
THE UNITED STATES MEDICAL AND SURGICAL JOURNAL, CHICAGO.
BOSTON JOURNAL OF CHEMISTRY.
JOURNAL OF APPLIED CHEMISTRY.
CANADA JOURNAL OF DENTAL SCIENCE, MONTREAL.
INDIANA JOURNAL OF MEDICINE.
MEDICAL AND SURGICAL REPORTER, SALEM, OREGON.
MISSOURI DENTAL JOURNAL, ST. LOUIS.
ECLECTIC MEDICAL JOURNAL, CINCINNATI.
THE SANITARAN, NEW YORK CITY.

### THE DENTAL COLLEGE

OF THE

### UNIVERSITY OF MICHIGAN.

The Fourth Annual Session of this Institution will commence on the 1st of October and close on the last Wednesday of March, thus making a course of six months. The regular course of instruction commences at once, and will proceed through the term, with the customary vacation.

The students in this department will receive instructions in Anatomy, Physiology, Pathology, Chemistry, Meteria Medica, Therapeutics and Surgery, from the professors of their respective branches in the *Department of Medicine and Surgery* of the University, when lectures commence, and continue the same as with the Dental College.

### FACULTY OF THE DENTAL DEPARTMENT.

J. B. ANGELL, LL. D., President.

J. TAFT, D. D. S., Professor of Principles and Practice of Operative Dentistry.

J. A. WATLING, D. D. S., Professor of Clinical and Mechanical Dentistry.

W. H. DONENEE, Demonstrator of Mechanical Dentistry.

It is desirable that students should be present at the opening of the session, as the regular course of instruction begins at that time. Seats in the lecture room are assigned by selection to students in the order of registration on the steward's books; and each student is expected to occupy during the session such seat as he may select. Students, on arriving in Ann Arbor, should call at the steward's office.

### CONDITIONS OF GRADUATION.

The candidate must be twenty-one years of age. He must furnish evidence of good moral character.

He must devote three years in the study of his profession. He must attend two full courses of lectures in the Dental College, or one course elsewhere and the last one here, and we recommend that he attend three courses regularly.

He must sustain an examination satisfactory to the Faculty in all the branches taught.

A graduate of the Medical College may enter the senior class, and, if found qualified, may graduate after one year has been devoted to the study of dentistry.

### FEES AND EXPENSES.

The fees, which must be paid in advance are as follows:

Matriculation Fee.—Residents of Michigan, $10.00; non-residents, $12.00.

Annual Dues.—Residents of Michigan, $20.00; non-residents, $25.00.

Graduation Fee.—For all alike, $10.00. The admission fee is paid but once, and entitles the students to the privileges of permanent membership in any department of the University. The annual tax is paid the first year and every year after.

For further particulars, address the Dean of the Dental College, Ann Arbor, Michigan.

Jan-6t. J. TAFT, Dean.

### THE DENTAL COLLEGE

OF THE

### UNIVERSITY OF MICHIGAN.

---

The Fourth Annual Session of this Institution will commence on the 1st of October and close on the last Wednesday of March, thus making a course of six months. The regular course of instruction commences at once, and will proceed through the term, with the customary vacation.

The students in this department will receive instructions in Anatomy, Physiology, Pathology, Chemistry, Meteria Medica, Therapeutics and Surgery, from the professors of their respective branches in the *Department of Medicine and Surgery* of the University, when lectures commence, and continue the same as with the Dental College.

### FACULTY OF THE DENTAL DEPARTMENT.

J. B. ANGELL, LL. D., President.

J. TAFT, D. D. S., Professor of Principles and Practice of Operative Dentistry.

J. A. WATLING, D. D. S., Professor of Clinical and Mechanical Dentistry.

W. H. DONENEE, Demonstrator of Mechanical Dentistry.

It is desirable that students shonld be present at the opening of the session, as the regular course of instruction begins at that time. Seats in the lecture room are assigned by selection to students in the order of registration on the steward's books; and each student is expected to occupy during the session such seat as he may select. Students, on arriving in Ann Arbor, should call at the steward's office.

### CONDITIONS OF GRADUATION.

The candidate must be twenty-one years of age. He must furnish evidence of good moral character.

He must devote three years in the study of his profession. He must attend two full cour es of lectures in the Dental College, or one course elsewhere and the last one here, and we recommend that he attend three courses regularly.

He must sustain an examination satisfactory to the Faculty in all the branches taught.

A graduate of the Medical College may enter the senior class, and, if found qualified, may graduate after one year has been devoted to the study of dentistry.

### FEES AND EXPENSES.

The fees, which must be paid in advance are as follows:

Matriculation Fee.—Residents of Michigan, $10.00; non-residents, $12.00.

Annual Dues.—Residents of Michigan, $20.00; non-residents, $25.00.d

Graduation Fee.—For all alike, $10.00. The admission fee is paid but once, and entitles the students to the privileges of permanent membership in any department of the University. The annual tax is paid the first year and every year after.

For further particulars, address the Dean of the Dental College, Ann Arbor, Michigan.

Jan-6t. J. TAFT Dean.

*American Dental Association*—Meets on the first Tuesday in August, 1878, at Niagara Falls. Pres. F. H. Rehwinkel, Chillicothe, Ohio; Cor. Sec. J. H. McQuillen, Philadelphia; Rec. Sec. M. S. Dean, Chicago.
*American Dental Convention*—Meets at Niagara Falls, first Tuesday in August, 1878. Pres. J. Taft, Cincinnati, Ohio; Rec. Sec. A. Tees, Philadelphia, Pa.

LIST OF SOCIETIES REPRESENTED IN THE AMERICAN DENTAL ASSOCIATION:

*American Academy of Dental Surgery*—Meets in New York 1878. Pres. Geo. H. Perine; Rec. Sec. N. M. Beckwith.
*American Academy of Dental Science*—Meets at Boston, Mass. Pres. Dr. Elisha Tucker, Boston ; Sec. E. B. Bradburn, of Boston ; Cor. Sec. Geo. T. Moffat.
*Baltimore Dental Society*—Pres. C. T. Brockett; Rec. Sec. F. F. Drew.
*Brooklyn Dental Society*—Meets semi-monthly. Pres. O. E. Hill; Rec. Sec. C. P. Crandell.
*Buffalo Dental Society*—Meets second Monday of each month, at Buffalo. Pres. S. A. Freeman; Rec. Sec. G. C. Daboll.
*Boston Dental Institute*—Meets first Monday of each month. Pres. D. G. Williams, Boston; Cor. Sec. D. S. Dickerman.
*California State Dental Association*—Meets first Tuesday in June, 1878. Pres. Wm. B. Kingsbury; Sec. H. J. Planteaux.
*Chicago Dental Society*—Meets monthy at Chicago. Pres. C. R. E. Koch; Rec. Sec. D. B. Freeman.
*Connecticut Valley Dental Association*—Meets semi-annually, second Tuesday of June and October. Pres. L. C. Laylor, Hartford, Conn.; Rec. Sec. C. T. Stockwell Springfield, Mass. Next Meeting in Springfield, Mass., October 17, 1877.
*Connecticut State Dental Society*—Pres. E. T. Payne; Rec. Sec. Geo. L. Parmelee, Hartford. Semi-annual meeting in October in Hartford.
*Charlestown Dental Association*—Meets second Tuesday of each month. Pres. T. F. Chupein; Sec. and Treas. B. A. Muckenfuss.
*Central Pennsylvania Dental Association*—Meets at Huntington, Jan. 1878. Pres. E. J. Green; Sec. W. B. Miller.
*Cincinnati Dental Society*—Meets first and third Tuesday of each month. Pres. A. Berry; Sec. A. Downing.
*Central Ohio Dental Society*—Meets at Crestline, on the second Monday in April, 1877. Pres. M. DeCamp; Sec. N. J. Cressinger.
*Dental Society of the State of Maryland and District of Columbia*—Meets annually. Next meeting at Deer Park, on the second Tuesday in August, 1877. Pres. R. F. Hunt, of Washington, D. C.; Sec. H. C, Thompson.
*Eastern Indiana Dental Association*—Meets semi-annually, first Tuesday in May, and last Tuesday in October; Pres. J. W. Ellis, New Castle; Sec. M. H. Chappell, Knightstown, Ind. Place of meeting, Cambridge City.
*East Tennessee Dental Association*—Meets annually. Next meeting will be held at Cleveland, Tenn., on the Third Wednesday of Sept, 1877. Pres. James Carson; Sec. J. U. Lee, of Chattanooga.
*Georgia State Dental Society*—July 30th, 1878. Pres. M. H. Thomas, Monroe, Ga. Rec. Sec. J. A. Chopple, Lagrange, Ga. Cor. Sec. Dr. M. S. Jobson, Perry, Ga.
*Harris Dental Association*—Meets quarterly. Next Meeting at Columbia, Pa., first Thursday in August. Pres. J. D. Heiges; Sec. W. N. Amer, Lancaster, Pa.
*Hudson Valley Dental Association*—Meets monthly at Troy, N. Y. Pres. L. C. Wheeler; Rec. Sec. S. G. Andres. Cor. Sec. S. D. French.
*Illinois State Dental Society*—Meets annually. Pres. C. R. E. Koch, Chicago; Sec. E. D. Swain, Chicago. Next place of meeting, Rockford, second Tuesday in May, 1878.
*Indiana State Dental Society*—Meets next year at Indianapolis, on the last Tuesday of June, 1878. Pres. T. S. Hacker, Indianapolis ; Sec. J. A. Turner, Franklin.
*Iowa State Dental Society*—Meets annually. Next meeting at Iowa City on the second Tuesday of May. Pres. G. S. Shattuck, Belle Plane; Rec. Sec. E. E. Hughes of Newton; Cor. Sec. I. P. Wilson, Burlington.
*Kentucky State Dental Asssciation*—Meets annually first Tuesday in June. Next meeting in Covington. Pres. A. W. Smith, of Richmond; Sec. F. Peabody of Louisville.
*Kansas State Dental Association*—Pres. J. D. Patterson; Sec. Wm. H. Shulze, of Atchison. Next meeting will be held at Lawrence, first Tuesday in May, 1878.
*Lake Erie Dental Association*—Meets annually. Pres. C. B. Ansart, Oil City, Penn. Sec. C. D. Elliot, Franklin, Penn. Next meeting first Tuesday of May, 1878.
*Mad River Dental Association*—Meets at Xenia on the first Tuesday in April, and October. Pres. R. Corson, Middletown; Sec. E. Betty, Xenia.
*Maine Dental Society*—Meets in August, at Thomaston. Pres. E. J. Roberts, Augusta. Sec C. E. Hussey, Biddeford.
*Massachusetts Dental Society*—Meets semi-annually. Pres. J. H. Kidder, of Lawrence ; Rec. Sec. Dwight M. Clapp, Boston; Cor. Sec. T. H. Chandler, Boston.
*Michigan State Dental Association*—Meets annually. Next meeting at Ann Arbor, March 26, 1878. Pres. R. G. Thomas, Detroit; Sec. and Treas. E. S. Holmes, Grand Rapids.
*Mississippi Valley Dental Association*—Meets annually at Cincinnati, on first Wednesday in March, 1878. Pres. A. O. Rawls, Lexington, Ky.; Rec. Sec. Wm. Taft, Cincinnati.

*Mississippi State Dental Association*—Will meet third Wednesday of August. 1878, at Winona. Pres. A. H. Hilzheim, Jackson; Sec. M. C. Marshall.

*Minnesota Dental Association*—Meets annually. Meets May 26. 1878 at Winona. Pres. W. F. Lewis; Sec. C. M. Bailey, Minneapolis.

*Missouri Dental Association*—Meets annually on the first Tuesday of June. Next meeting at Sweet Springs. Pres. G. W. Tindall; Sec. W. H. Eames, of St. Louis.

*Missouri Valley Dental Association*—Meets on the fourth Tuesday in July, 1876, in Omaha, Neb. Pres. J. W. Chadduck; Sec. and Treas. F. M. Shrirer, Tabor, Iowa.

*Merrimack Valley Dental Association*—Meets on the 3d Thursday of May, 1878, in Boston. Pres. C. G. Davis, New Bedford Mass.; Rec. Sec. W. E. Riggs, Lawrence, Mass.; Cor. Sec. D. W. Edgerly, Farmington, N. H.

*Memphis Dental Society*—Meets on the first Tuesday of each month. Pres. W. M. Arrington; Sec. V. F. Elliot.; Treas. C. L. Harris.

*Nashville Dental Association*—Meets on the second Tuesday of each month. Pres. R. R. Freeman; Sec. L. G. Noel, Nashville.

*Nebraska State Dental Society*—Meets annually. Next meeting July 23, 1878, at Fremont. Pres. S. H. King, Lincoln. Sec. W. F. Roseman, Fremont.

*New Jersey State Dental Society*—Meets annually. Next meeting at Long Branch, on the second Tuesday in July, 1877. Pres. T. B. Welch, Newark; Sec. C. A. Meeker, Newark.

*New Hampshire Dental Society*—Pres. D. W. Eagerly, Farmington; Sec. C. W. Clerment, Manchester.

*New Haven Dental Society*— Meets on the first Wednesday of each month at New Haven. Pres. J. T. Metcalf; Rec. and Cor. Sec. C. L. Smith, New Haven.

*New Orleans Dental Society*—Meets on the first Thursday of each month. Pres. W. S. Chandler; Rec. Sec. F. J. Knapp.

*Northern Ohio Dental Association*—Meets annually. Next meeting at Elyria, on the second Tuesday in May, 1878. Pres. J. W. Lyder, Akron, O. Sec. J. F. Siddall, Oberlin.

*Northern Iowa Dental Association*—Meets annually, on the second Tuesday in June, 1873. Next Meeting at Waterloo. Pres. J. M. Abbot, Lancaster; Rec. Sec. A. V. Eaton, Anamosa; Cor. Sec. A. R. Mason, Waterloo.

*North Carolina Dental Association*—Meets annually. Next meeting at Charlotte, first Tuesday in Sept., 1878. Pres. J. W. Hunter, Salem; Sec. J. H. Crawford, Raleigh.

*North Carolina State Dental Society*—Meets annually. Next meeting at Raleigh, first Wednesday Sept., 1877. Pres. V. E. Turner; Sec. D. A. Robinson.

*Ohio College Dental Association*—Meets annually, March 4, 1879 at Cincinnati. Pres. F. A. Hunter, Mo; Rec. Sec. H. A. Smith, of Cincinnati.

*Ohio State Dental Association*—Meets annually at Columbus, first Tuesday of December, 1878. Pres. J. C. Whinnery, Salem; Rec. Sec. A. F. Eminger, Columbus.

*Odontological Society of Pennsylvania*—Meets on the first Wednesday of each month, at 8 o'clock p. m., in the Philadelphia Dental College. Pres. F. M. Dixon; Rec. Sec. E. L. Hewitt.

*Odontological Society of New York City*—Meets the second Tuesday of each month. Pres. A. L. Northrup; Rec. Sec. Wm. Jarvie, Jr.

*Old Colony Dental Association*—Meets quarterly. Pres. E. L. Hathaway; Rec. Sec. L. W. Puffer, North Bridgewater, Mass.

*Ohio Valley Dental Association*—Meets annually, in N. E. Ky., this year in Paris, Ky., Tuesday, September 9th.

*Oregon State Dental Society*—Meets annually. Pres. J. R. Cardwell, Portland; Rec. Sec. G. N. Chance, Portland.

*Pennsylvania State Dental Society*—Meets next in Bedford, Tuesday July, 1878. Pres. J. S. King; Rec. Sec. S. H. Guilford; Cor. Sec. M. H. Webb.

*Pennsylvania Association of Dental Surgeons*—Meets monthly in Philadelphia. Pres Robert Henry; Sec. Jos. Pettit.

*Pittsburg Dental Society*—Meets second Tuesday of each month. Pres. H. W. Arthur, of Alleghany; Sec. E. C. Diehl, Pittsburg.

*San Francisco Dental Association*—Meets monthly. Pres. Alex. Warner. Vice Pres. R. F. Rall; Cor. Sec. S. E. Goe; Rec. Sec. L. L. Dunbar.

*St. Louis Dental Society*—Meets monthly. Pres. A. H. Fuller; Sec. N. C. Stark.

*Susquehanna Dental Association*—Meets annually. Pres. J. N. Johnson; Rec. Sec. E. B. Long. Meets November 14, 1877, at Wilkesbarre.

*Society of Dental Surgeons of the City of New York*—Meets semi-monthly in New York. Pres. B. F. Franklin; Sec. J. S. Latimer; Cor. Sec. E. H. Burras.

*Southern Dental Association*—Meets annually. Next meeting at Deer Park, August 14, 1877. Pres. J. S. Cobb, Nashville, Tenn.; Rec. Sec. E. E. Chisholm, Tuscaloosa, Ala.

*South Carolina State Dental Association*.—Meets annually. Pres. G. F. S. Wright, Columbia; Sec. T. F. Cheupein, Charleston; Cor. Sec. J. S. Thompson, Abbeville. Next meeting at Columbia, the first Tuesday of June, 1877.

*Tennesee Dental Association*—Meets semi-annually. Next meeting at Nashville, fourth Wednesday in June, 1878. Pres. L. G. Noel; Rec. Sec. H. E. Beach; Cor. Sec. J. C. Ross, Nashville.

*Vermont State Dental Society*—Meets annually.

*Virginia State Dental Association*—Meets in Richmond, December the 14. Pres. W. W. H. Thackston, Farmville; Sec. G. F. Keesee, Richmond.

*Washington City Dental Society*—Meets monthly. Pres. J. C. Smith; Sec. J. B. TenEyck.

*Wisconsin State Dental Association*—Meets annually. Next meeting at Milwaukee, second Tuesday of July, 1877. Pres. R. S. Wells, Sparta; Sec. M. T. Moore, LaCrosse.

### State and District Dental Societies of State of New York.

*New York State Dental Society*—Meets at Albany on the second Wednesday of July next. Pres. W. C. Barrett, Warsaw, N. York.; Cor. Sec. S. B. Palmer; Rec. Sec. S. S. A. Freeman, Buffalo.

*First District*—Meets second and fourth Wednesday evenings of each month. Annual meeting in New York, first Tuesday in June. Pres. J. S. Latimer, New York; Rec. Sec. F. M. Odell, New York.

*Second District*—Annual meeting in Brooklyn, first Monday in April. Pres. C. D. Cook; Rec. Sec. G. L. Mason, Brooklyn; Cor. Sec. W. S. Elliott. The meetings are held quarterly, second Thursday in January, April, July, and October.

*Third District*—Meets semi-annually on the second Tuesday in January and July. Annual meeting at Albany on the second Tuesday in January. Pres. S. P. Welsh.

*Fourth District*—Meets semi-annually. Meets annually at Saratoga on second Tuesday in June. Pres. C. A. Elmore, Ft. Edward; Rec. Sec. E. S. Pearsall, Saratoga.

*Fifth District*—Meets semi-annually. Annual meeting at Syracuse, second Tuesday in June. Pres. F. D. Nellis, Syracuse; Rec. Sec. J. S. Marshall, Syracuse; Cor. Sec. A. B. Cowles, Rome.

*Sixth District*—Meets semi-annually. Annual meeting at Binghampton, on second Tuesday in June. Pres. Frank B. Darby, Oswego; Rec. Sec. F. E. Perry, Norwich; Cor. Sec. A. M. Holmes, Morrisville.

*Seventh District*—Meets semi-annually. Annual meeting at Rochester, first Tuesday in June. Pres. H. S. Miller; Rec. Sec. J. E. Line, Rochester.

*Eighth District*—Meets semi-annually. Annual meeting on second Tuesday in May. Pres. G. C. Daboll; Rec. Sec. S. A. Freeman; Cor. Sec. T. G. Lewis, Buffalo.

*The Seventh and Eighth District Societies*—Hold a union meeting on the last Tuesday of October, alternately at Buffalo and Rochester.

*The Ohio State Board of Dental Examiners* will meet in Columbus on the first Tuesday of December, 1878, at 12 o'clock, M. Pres. J. Taft; Sec. W. P. Horton.

### EXCHANGES.

ST. LOUIS MEDICAL AND SURGICAL JOURNAL.
BOSTON MEDICAL AND SURGICAL JOURNAL.
THE PACIFIC MEDICAL AND SURICAL JOURNAL, SAN FRANCISCO.
PHILADELPHIA MEDICAL AND SURGICAL JOURNAL.
CINCINNATI MEDICAL ADVANCE.
CINCINNATI LANCET AND OBSERVER.
DENTAL COSMOS, PHILADELPHIA.
LADIES' REPOSITORY, CINCINNATI.
CHICAGO MEDICAL EXAMINER.
THE DETROIT REVIEW OF MEDICINE AND PHARMACY.
MEDICAL RECORD, N. Y.
NASHVILLE JOURNAL OF MEDICINE AND SURGERY.
AMERICAN JOURNAL OF DENTAL SCIENCE, BALTIMORE.
THE UNITED STATES MEDICAL AND SURGICAL JOURNAL, CHICAGO.
BOSTON JOURNAL OF CHEMISTRY.
JOURNAL OF APPLIED CHEMISTRY.
CANADA JOURNAL OF DENTAL SCIENCE, MONTREAL.
INDIANA JOURNAL OF MEDICINE.
MEDICAL AND SURGICAL REPORTER, SALEM, OREGON
MISSOURI DENTAL JOURNAL, ST. LOUIS.
ECLECTIC MEDICAL JOURNAL, CINCINNATI.
THE SANITARAN, NEW YORK CITY.

### THE DENTAL COLLEGE

OF THE

### UNIVERSITY OF MICHIGAN.

The Fourth Annual Session of this Institution will commence on the 1st of October and close on the last Wednesday of March, thus making a course of six months. The regular course of instruction commences at once, and will proceed through the term, with the customary vacation.

The students in this department w ll receive instructions in Anatomy, Physiology, Pathology, Chemistry, Meteria Medica, Therapeutics and Surgery, from the professors of their respective branches in the *Department of Medicine and Surgery* of the University, when lectures commence, and continue the same as with the Dental College.

### FACULTY OF THE DENTAL DEPARTMENT.

J. B. ANGELL, LL. D., President.

J. TAFT, D. D. S., Professor of Principles and Practice of Operative Dentistry.

J. A. WATLING, D. D. S., Professor of Clinical and Mechanical Dentistry.

W. H. DONENEE, Demonstrator of Mechanical Dentistry.

It is desirable that students should be present at the opening of the session, as the regular course of instruction begins at that time. Seats in the lecture room are assigned by selection to students in the order of registration on the steward's books; and each student is expected to occupy during the session such seat as he may select. Studes on arriving in Ann Arbor, should call at the steward's office.

### CONDITIONS OF GRADUATION.

The candidate must be twenty-one years of age. He must furnish dence of good moral character.

He must devote three years in the study of his profession. He must attend two full cour-es of lectures in the Dental College, or one course elsewhere and the last one here, and we recommend that he attend three courses regularly.

He must sustain an examination satisfactory to the Faculty in all the branches taught.

A graduate of the Medical College may enter the senior class, and, if found qualified, may graduate after one year has been devoted to the study of dentistry.

### FEES AND EXPENSES.

The fees, which must be paid in advance are as follows:

Matriculation Fee.—Residents of Michigan, $10.00; non-residents, $12.00.

Annual Dues.—Residents of Michigan, $20.00; non-residents, $25.00.d

Graduation Fee.—For all alike, $10.00. The admission fee is paidbut once, and entitles the students to the privileges of permanent membership in any department of the University. The annual tax is paid the first year and every year after.

For further particulars, address the Dean of the Dental College, Ann Ar bor, Michigan.

Jan-6t. J. TAFT, Dean.

### THE DENTAL COLLEGE

OF THE

### UNIVERSITY OF MICHIGAN.

---

The Fourth Annual Session of this Institution will commence on the 1st of October and close on the last Wednesday of March, thus making a course of six months. The regular course of instruction commences at once, and will proceed through the term, with the customary vacation.

The students in this department will receive instructions in Anatomy, Physiology, Pathology, Chemistry, Meteria Medica, Therapeutics and Surgery, from the professors of their respective branches in the *Department of Medicine and Surgery* of the University, when lectures commence, and continue the same as with the Dental College.

### FACULTY OF THE DENTAL DEPARTMENT.

J. B. ANGELL, LL. D., President.

J. TAFT, D. D. S., Professor of Principles and Practice of Operative Dentistry.

J. A. WATLING, D. D. S., Professor of Clinical and Mechanical Dentistry.

W. H. DONENEE, Demonstrator of Mechanical Dentistry.

It is desirable that students should be present at the opening of the session, as the regular course of instruction begins at that time. Seats in the lecture room are assigned by selection to students in the order of registration on the steward's books; and each student is expected to occupy during the session such seat as he may select. Students on arriving in Ann Arbor, should call at the steward's office.

### CONDITIONS OF GRADUATION.

The candidate must be twenty-one years of age. He must furnish evidence of good moral character.

He must devote three years in the study of his profession. He must attend two full courses of lectures in the Dental College, or one course elsewhere and the last one here, and we recommend that he attend three courses regularly.

He must sustain an examination satisfactory to the Faculty in all the branches taught.

A graduate of the Medical College may enter the senior class, and if found qualified, may graduate after one year has been devoted to the study of dentistry.

### FEES AND EXPENSES.

The fees, which must be paid in advance are as follows:

Matriculation Fee.—Residents of Michigan, $10.00; non-residents, $12.00.

Annual Dues.—Residents of Michigan, $20.00; non-residents, $25.00.d

Graduation Fee.—For all alike, $10.00. The admission fee is paid but once, and entitles the students to the privileges of permanent membership in any department of the University. The annual tax is paid the first year and every year after.

For further particulars, address the Dean of the Dental College, Ann Arbor, Michigan.

Jan-6t. J. TAFT, Dean.

### THE DENTAL COLLEGE

OF THE

### UNIVERSITY OF MICHIGAN.

---

The Fourth Annual Session of this Institution will commence on the 1st of October and close on the last Wednesday of March, thus making a course of six months. The regular course of instruction commences at once, and will proceed through the term, with the customary vacation.

The students in this department will receive instructions in Anatomy, Physiology, Pathology, Chemistry, Meteria Medica, Therapeutics and Surgery, from the professors of their respective branches in the *Department of Medicine and Surgery* of the University, when lectures commence, and continue the same as with the Dental College.

### FACULTY OF THE DENTAL DEPARTMENT.

J. B. ANGELL, LL. D., President.

J. TAFT, D. D. S., Professor of Principles and Practice of Operative Dentistry.

J. A. WATLING, D. D. S., Professor of Clinical and Mechanical Dentistry.

W. H. DONENEE, Demonstrator of Mechanical Dentistry.

It is desirable that students should be present at the opening of the session, as the regular course of instruction begins at that time. Seats in the lecture room are assigned by selection to students in the order of registration on the steward's books; and each student is expected to occupy during the session such seat as he may select. Students on arriving in Ann Arbor, should call at the steward's office.

### CONDITIONS OF GRADUATION.

The candidate must be twenty-one years of age. He must furnish evidence of good moral character.

He must devote three years in the study of his profession. He must attend two full courses of lectures in the Dental College, or one course elsewhere and the last one here, and we recommend that he attend three courses regularly.

He must sustain an examination satisfactory to the Faculty in all the branches taught.

A graduate of the Medical College may enter the senior class, and if found qualified, may graduate after one year has been devoted to the study of dentistry.

### FEES AND EXPENSES.

The fees, which must be paid in advance are as follows:

MATRICULATION FEE.—Residents of Michigan, $10.00; non-residents, $12.00.

ANNUAL DUES.—Residents of Michigan, $20.00; non-residents, $25.00.d

GRADUATION FEE.—For all alike, $10.00. The admission fee is paid but once, and entitles the students to the privileges of permanent membership in any department of the University. The annual tax is paid the first year and every year after.

For further particulars, address the Dean of the Dental College, Ann Arbor, Michigan.

Jan-6t. J. TAFT, DEAN.

### *OHIO COLLEGE OF DENTAL SURGERY.*

COLLEGE STREET.

The Thirty-third Annual Session 1878-9.

### FACULTY.

JAMES TAYLOR, M. D., D. D. S,

Principles and Practice, and Dental Hygiene.

WM. CLENDENNIN, M. D.,

Anatomy.

J. S. CASSIDY, M. D., D. D. S.,
Chemistry and Materia Medica.

J. L. CILLEY, M. D.,
Physiology and Histology.

A. O. RAWLS, D. D. S.,

Pathology and Therapeutics.

H. A. SMITH, D. D. S,

Clinical Dentistry.

J. R. CLAYTON, D. D. S.,
Mechanical Dentistry and Microscopy.

---

### SPECIAL PROFESSORS.

F. H. REHWINKEL, M. D., D. D. S.,
Oral Surgery.

G. W. KEELY, D. D. S.,
Cause and Management of Irregularities of the Teeth.

---

C. I. KEELY, D. D. S..
Demonstrator of Operative Dentistry.

FRANK BELL, D. D. S.,
Demonstrator of Continuous Gum Work.

S. WARDELL, D. D. S.,
Demonstrator of Carving Teeth.

ALEX. BROWN, D. D. S.,

Assistant to Chair of Chemistry.

E. G. BETTY, D. D. S., Demonstrator of Anatomy.

---

### CLINICAL INSTRUCTORS.

DR. S. B. BROWN, DR. F. A. HUNTER, DR. A. W. SMITH, DR. D. W. CLANCEY, DR. J. E CRAVENS, DR. J. K. JAMISON, DR. J. I. TAYLOR, DR. F. PEABODY, DR. C. R. TAFT, DR. A. F. EMMINGER, DR. W. S. HOW.

This institution being strictly a *Dental College*, the lectures in all the branches taught, Anatomy, Physiology, Pathology, Therapeutics, and Materia Medica included, are arranged with the view to qualify the student to intelligently and successfully practice his profession.

The College Building was designed, erected, and is exclusively used for a Dental School. The lecture and clinical rooms are therefore well adapted for the uses to which they are put.

### TERMS OF GRADUATION.

A candidate for graduation must be twenty-one years of age. He must have attended two courses of lectures in a Dental or Medical College, the last of which must be in this institution. A reputable dentist in actual practice five years, inclusive of the term of pupilage, may be examined for the degree of D. D. S., after attending one full course of lectures.

### FEES.

| | |
|---|---|
| Matriculation Fee (but once) | $ 5 00 |
| Professor's Tickets for one session | 75 00 |
| Demonstrator's Ticket (for Anatomy) | 5 00 |
| Diploma Fee | 20 00 |

It is desirable that the students should matriculate and be in attendance promptly at the beginning of the term. The term begins the 1st of November, and continues sixteen weeks.

Students coming to the city should call on the Dean. Good board can be had for $3.00 to $5.00 per week.

For further information, address

***H. A. SMITH, Dean,***
286 Race St., CINCINNATI.

### *OHIO COLLEGE OF DENTAL SURGERY.*

COLLEGE STREET.

The Thirty-third Annual Session 1878-9.

### FACULTY.

JAMES TAYLOR, M. D., D. D. S,

Principles and Practice, and Dental Hygiene.

WM. CLENDENNIN, M. D.,

Anatomy.

J. S. CASSIDY, M. D., D. D. S.,
Chemistry and Materia Medica.

J. L. CILLEY, M. D.,
Physiology and Histology.

A. O. RAWLS, D. D. S.,

Pathology and Therapeutics.

H. A. SMITH, D. D. S,

Clinical Dentistry.

J. R. CLAYTON, D. D. S.,
Mechanical Dentistry and Microscopy.

---

### SPECIAL PROFESSORS.

F. H. REHWINKEL, M. D., D. D. S.,
Oral Surgery.

G. W. KEELY, D. D. S.,
Cause and Management of Irregularities of the Teeth.

---

C. I. KEELY, D. D. S..
Demonstrator of Operative Dentistry.

FRANK BELL, D. D. S.,
Demonstrator of Continuous Gum Work.

S. WARDELL, D. D. S.,
Demonstrator of Carving Teeth.

ALEX. BROWN, D. D. S.,

Assistant to Chair of Chemistry.

E. G. BETTY, D. D. S., Demonstrator of Anatomy.

---

### CLINICAL INSTRUCTORS.

DR. S. B. BROWN.

DR. F. A. HUNTER,

DR. A. W. SMITH,

DR. D. W. CLANCEY,

DR. J. E. CRAVENS,

DR. J. K. JAMISON,

DR. J. I. TAYLOR,

DR. F. PEABODY,

DR. C. R. TAFT,

DR. A. F. EMMINGER,

DR. W. S. HOW.

This institution being strictly a *Dental College*, the lectures in all the branches taught, Anatomy, Physiology, Pathology, Therapeutics, and Materia Medica included, are arranged with the view to qualify the student to intelligently and successfully practice his profession.

The College Building was designed, erected, and is exclusively used for a Dental School. The lecture and clinical rooms are therefore well adapted for the uses to which they are put.

### TERMS OF GRADUATION.

A candidate for graduation must be twenty-one years of age. He must have attended two courses of lectures in a Dental or Medical College, the last of which must be in this institution. A reputable dentist in actual practice five years, inclusive of the term of pupilage, may be examined for the degree of D. D. S., after attending one full course of lectures.

### FEES.

| | |
|---|---|
| Matriculation Fee (but once) - - - - - - | $ 5 00 |
| Professor's Tickets for one session - - - - - - | 75 00 |
| Demonstrator's Ticket (for Anatomy) - - - - - | 5 00 |
| Diploma Fee - - - - - - - - - | 20 00 |

It is desirable that the students should matriculate and be in attendance promptly at the beginning of the term. The term begins the 1st of November, and continues sixteen weeks.

Students coming to the city should call on the Dean. Good board can be had for $3.00 to $5.00 per week.

For further information, address

***H. A. SMITH, Dean,***
286 Race St., CINCINNATI.

Aug-5

### *OHIO COLLEGE OF DENTAL SURGERY.*

COLLEGE STREET.

The Thirty-third Annual Session 1878-9.

### FACULTY.

JAMES TAYLOR, M. D., D. D. S.,

Principles and Practice, and Dental Hygiene.

WM. CLENDENNIN, M. D.,

Anatomy.

J. S. CASSIDY, M. D., D. D. S.,
Chemistry and Materia Medica.

J. L. CILLEY, M. D.,
Physiology and Histology.

A. O. RAWLS, D. D. S.,

Pathology and Therapeutics.

H. A. SMITH, D. D. S ,

Clinical Dentistry.

J. R. CLAYTON, D. D. S.,
Mechanical Dentistry and Microscopy.

### SPECIAL PROFESSORS.

F. H. REHWINKEL, M. D., D. D. S.,
Oral Surgery.

G. W. KEELY, D. D. S.,
Cause and Management of Irregularities of the Teeth.

C. I. KEELY, D. D. S.,
Demonstrator of Operative Dentistry.

FRANK BELL, D. D. S.,
Demonstrator of Continuous Gum Work.

S. WARDELL, D. D. S.,
Demonstrator of Carving Teeth.

ALEX. BROWN, D. D. S.,

Assistant to Chair of Chemistry.

E. G. BETTY, D. D. S., Demonstrator of Anatomy.

### CLINICAL INSTRUCTORS.

DR. S. B. BROWN.

DR. F. A. HUNTER,

DR. A. W. SMITH,

DR. D. W. CLANCEY,

DR. J. E. CRAVENS,

DR. J. K. JAMISON,

DR. J. I. TAYLOR,

DR. F. PEABODY,

DR. C. R. TAFT,

DR. A. F. EMMINGER,

DR. W. S. HOW.

This institution being strictly a *Dental College*, the lectures in all the branches taught, Anatomy, Physiology, Pathology, Therapeutics, and Materia Medica included, are arranged with the view to qualify the student to intelligently and successfully practice his profession.

The College Building was designed, erected, and is exclusively used for a Dental School. The lecture and clinical rooms are therefore well adapted for the uses to which they are put.

### TERMS OF GRADUATION.

A candidate for graduation must be twenty-one years of age. He must have attended two courses of lectures in a Dental or Medical College, the last of which must be in this institution. A reputable dentist in actual practice five years, inclusive of the term of pupilage, may be examined for the degree of D. D. S., after attending one full course of lectures.

### FEES.

| | |
|---|---|
| Matriculation Fee (but once) - - - - - - - | $ 5 00 |
| Professor's Tickets for one session - - - - - - | 75 00 |
| Demonstrator's Ticket (for Anatomy) - - - - - | 5 00 |
| Diploma Fee - - - - - - - - - - | 20 00 |

It is desirable that the students should matriculate and be in attendance promptly at the beginning of the term. The term begins the 1st of November, and continues sixteen weeks.

Students coming to the city should call on the Dean. Good board can be had for $3.00 to $5.00 per week.

For further information, address

***H. A. SMITH, Dean,***
286 Race St., CINCINNATI.

Aug-78

### *OHIO COLLEGE OF DENTAL SURGERY.*

COLLEGE STREET.

The Thirty-third Annual Session 1878-9.

### FACULTY.

JAMES TAYLOR, M. D., D. D. S.,

Principles and Practice, and Dental Hygiene.

WM. CLENDENNIN, M. D.,

Anatomy.

J. S. CASSIDY, M. D., D. D. S.,
Chemistry and Materia Medica.

J. L. CILLEY, M. D.,
Physiology and Histology.

A. O. RAWLS, D. D. S.,

Pathology and Therapeutics.

H. A. SMITH, D. D. S ,

Clinical Dentistry.

J. R. CLAYTON, D. D. S.,
Mechanical Dentistry and Microscopy.

### SPECIAL PROFESSORS.

F. H. REHWINKEL, M. D., D. D. S.,
Oral Surgery.

G. W. KEELY, D. D. S.,
Cause and Management of Irregularities of the Teeth.

C. I. KEELY, D. D. S..
Demonstrator of Operative Dentistry.

FRANK BELL, D. D. S.,
Demonstrator of Continuous Gum Work.

S. WARDELL, D. D. S.,
Demonstrator of Carving Teeth.

ALEX. BROWN, D. D. S.,

Assistant to Chair of Chemistry.

E. G. BETTY, D. D. S., Demonstrator of Anatomy.

### CLINICAL INSTRUCTORS.

DR. S. B. BROWN.

DR. F. A. HUNTER,

DR. A. W. SMITH,

DR. D. W. CLANCEY,

DR. J. E. CRAVENS,

DR. J. K. JAMISON,

DR. J. I. TAYLOR,

DR. F. PEABODY,

DR. C. R. TAFT,

DR. A. F. EMMINGER,

DR. W. S. HOW.

This institution being strictly a *Dental College*, the lectures in all the branches taught, Anatomy, Physiology, Pathology, Therapeutics, and Materia Medica included, are arranged with the view to qualify the student to intelligently and successfully practice his profession.

The College Building was designed, erected, and is exclusively used for a Dental School. The lecture and clinical rooms are therefore well adapted for the uses to which they are put.

### TERMS OF GRADUATION.

A candidate for graduation must be twenty-one years of age. He must have attended two courses of lectures in a Dental or Medical College, the last of which must be in this institution. A reputable dentist in actual practice five years, inclusive of the term of pupilage, may be examined for the degree of D. D. S., after attendng one full course of lectures.

### FEES.

| | |
|---|---|
| Matriculation Fee (but once) - - - - - - - | $ 5 00 |
| Professor's Tickets for one session - - - - - - - | 75 00 |
| Demonstrator's Ticket (for Anatomy) - - - - - - | 5 00 |
| Diploma Fee - - - - - - - - - - | 20 00 |

It is desirable that the students should matriculate and be in attendance promptly at the beginning of the term. The term begins the 1st of November, and continues sixteen weeks.

Students coming to the city should call on the Dean. Good board can be had for $3.00 to $5.00 per week.

For further information, address

***H. A. SMITH, Dean,***

Aug-78 286 Race St., CINCINNATI.

### *OHIO COLLEGE OF DENTAL SURGERY.*

COLLEGE STREET.

The Thirty-third Annual Session 1878-9.

### FACULTY.

JAMES TAYLOR, M. D., D. D. S.,

Principles and Practice, and Dental Hygiene.

WM. CLENDENNIN, M. D.,

Anatomy.

J. S. CASSIDY, M. D., D. D. S.,
Chemistry and Materia Medica.

J. L. CILLEY, M. D.,
Physiology and Histology.

A. O. RAWLS, D. D. S.,

Pathology and Therapeutics.

H. A. SMITH, D. D. S,

Clinical Dentistry.

J. R. CLAYTON, D. D. S.,
Mechanical Dentistry and Microscopy.

### SPECIAL PROFESSORS.

F. H. REHWINKEL, M. D., D. D. S.,
Oral Surgery.

G. W. KEELY, D. D. S.,
Cause and Management of Irregularities of the Teeth.

C. I. KEELY, D. D. S.,
Demonstrator of Operative Dentistry.

FRANK BELL, D. D. S.,
Demonstrator of Continuous Gum Work.

S. WARDELL, D. D. S.,
Demonstrator of Carving Teeth.

ALEX. BROWN, D. D. S.,

Assistant to Chair of Chemistry.

E. G. BETTY, D. D. S., Demonstrator of Anatomy.

### CLINICAL INSTRUCTORS.

DR. S. B. BROWN, DR. F. A. HUNTER, DR. A. W. SMITH, DR. D. W. CLANCEY, DR. J. E. CRAVENS, DR. J. K. JAMISON, DR. J. I. TAYLOR, DR. F. PEABODY, DR. C. R. TAFT, DR. A. F. EMMINGER, DR. W. S. HOW.

This institution being strictly a *Dental College*, the lectures in all the branches taught, Anatomy, Physiology, Pathology, Therapeutics, and Materia Medica included, are arranged with the view to qualify the student to intelligently and successfully practice his profession.

The College Building was designed, erected, and is exclusively used for a Dental School. The lecture and clinical rooms are therefore well adapted for the uses to which they are put.

### TERMS OF GRADUATION.

A candidate for graduation must be twenty-one years of age. He must have attended two courses of lectures in a Dental or Medical College, the last of which must be in this institution. A reputable dentist in actual practice five years, inclusive of the term of pupilage, may be examined for the degree of D. D. S., after attending one full course of lectures.

### FEES.

| | |
|---|---|
| Matriculation Fee (but once) | $ 5 00 |
| Professor's Tickets for one session | 75 00 |
| Demonstrator's Ticket (for Anatomy) | 5 00 |
| Diploma Fee | 20 00 |

It is desirable that the students should matriculate and be in attendance promptly at the beginning of the term. The term begins the 1st of November, and continues sixteen weeks.

Students coming to the city should call on the Dean. Good board can be had for $3.00 to $5.00 per week.

For further information, address

***H. A. SMITH, Dean,***
286 Race St., CINCINNATI.

Aug-6t

### CONTENTS.

### *THE DENTAL REGISTER,*

A MONTHLY MAGAZINE DEVOTED TO THE INTERESTS OF

### THE DENTAL PROFESSION.

*Terms Reduced to $2 50 per Year. Single Copies 25c.*

### *EDITED BY PROF. J. TAFT, D. D. S.,*

AND PUBLISHED BY

SPENCER & CROCKER, Ohio Dental Depot.

The volume commences in January and closes in December.

The REGISTER being issued monthly is always fresh, therefore Indispensable to the practitioner who desires to keep posted up with the new inventions and improvements which are daily developed. Neither expense nor effort will be spared to give value and variety to its contents. Articles will be illustrated with well executed engravings whenever they will add to the interest or assist to explanation.

Its extensive circulation and frequency of issue make it one of the BEST DENTAL ADVERTISING MEDIUMS in the United States.

All subscriptions must begin with January or July.

Local or special notices, five lines of less, will be inserted for $3.00 each insertion; over five lines, 50 cts. per line.

All advertising bills payable in advance, or at furthest, after the issue of the first number containing the advertisement. All transient advertisements to be paid for in advance.

☞CONTRIBUTIONS SOLICITED.

Exclusively Editorial Communications should be addressed to Editor.

The latest number of the REGISTER may be ordered with or without other goods at any time, and all letters should be addressed to

**SPENCER & CROCKER, Ohio Dental Depot, Cincinnati, O.**

